# Targeted killing of myofibroblasts by biosurfactant di-rhamnolipid suggests a therapy against scar formation

**DOI:** 10.1038/srep37553

**Published:** 2016-11-30

**Authors:** Chong Shen, Lifang Jiang, Huawei Shao, Chuangang You, Guoliang Zhang, Sitong Ding, Tingwei Bian, Chunmao Han, Qin Meng

**Affiliations:** 1 College of Chemical and Biological Engineering, Zhejiang University, Hangzhou, PR China; 2 Department of Burns & Wound Care Centre, Second Affiliated Hospital of Zhejiang University, College of Medicine, Hangzhou, PR China; 3 Ocean College, Zhejiang University of Technology, Hangzhou, PR China

## Abstract

Pathological myofibroblasts are often involved in skin scarring via generating contractile force and over-expressing collagen fibers, but no compound has been found to inhibit the myofibroblasts without showing severe toxicity to surrounding physiological cells. Here we report that di-rhamnolipid, a biosurfactant secreted by *Pseudomonas aeruginosa*, showed potent effects on scar therapy via a unique mechanism of targeted killing the myofibroblasts. In cell culture, the fibroblasts-derived myofibroblasts were more sensitive to di-rhamnolipid toxicity than fibroblasts at a concentration-dependent manner, and could be completely inhibited of their specific functions including α-SMA expression and collagen secretion/contraction. The anti-fibrotic function of di-rhamnolipid was further verified in rabbit ear hypertrophic scar models by presenting the significant reduction of scar elevation index, type I collagen fibers and α-SMA expression. In this regard, di-rhamnolipid treatment could be suggested as a therapy against skin scarring.

Myofibroblast, a specialized contractile fibroblast, plays the essential role in forming pathological skin scars in wound healing^1^. Transformed from fibroblasts under stimulation of inflammatory cytokines (eg, TGF-β1) and mechanical stress, myofibroblasts express contractile apparatus such as α-smooth muscle actin (α-SMA) and secrete collagen propolypeptides to repair the open wound^1^. When a normal healing wound closes, myofibroblasts are anticipated to disappear via apoptosis. Unexpectedly, myofibroblasts continue to proliferate and remodel collagen fibers in process of scarring. The over-accumulated and contracted collagen fibers then elevate the skin surface and enhance the tissue stiffness, leading to the hypertrophic scars or keloids in clinic^1^. Such scars usually bring much pain to patients for the impaired functionality of the pediatric tissues^2^ and even devastating psychosocial effects^3^.

The two main strategies used in clinical scar therapy are either inhibiting all skin cells or blocking the transformed pathway of myofibroblasts, but they have their limitations. Corticosteroids (eg, triamcinolone acetonide) and chemotherapeutic drugs (eg, 5-fluorouracil) in clinical practice halted the mitosis of all skin cells including physiological fibroblasts, endothelial cells and keratinocytes^3^,^4^,^5^, so that some side effects such as burning and irritation could occur on skin^5^. The two nucleotide molecules, EXC-001 (an antisense oligonucleotide, Pfizer) and RXI-109 (a small interfering RNA, RXi Pharmaceuticals) which have entered into phase II and III of clinical trials, could block the transdifferentiation of myofibroblasts via the connective tissue growth factor (CTGF) pathway^3^. Unfortunately, such nucleotides only performed moderate effect against scar elevation and did not benefit to wound healing and collagen reorganization^6^. Moreover, nucleotide drugs are supposed to be expensive and susceptible to nuclease in environment^7^. Hence, specifically killing myofibroblasts without harming the surrounding physiologic cells has been proposed as a novel strategy in future therapy of scar formation due to its high efficiency and safety^8^. However, no such compound has ever been reported.

Di-rhamnolipid (RHA), a biosurfactant secreted by *Pseudomonas aeruginosa*, has been previously found to show anti-fibrotic functions by reducing collagen content in burn wounds of rats^9^, but its mechanism was incorrectly interpreted as inhibiting the proliferation of dermal fibroblasts^9^,^10^. In this paper, we report that RHA at 10–30 mg/L shows a unique effect on selectively killing myofibroblasts without causing significant toxicity on fibroblasts, suggesting a completely different mechanism from currently prescribed drugs in scar therapy. For confirming its anti-scarring effect, rabbit ear hypertrophic scar models are used in our study instead of previously reported burn wounded rats^9^ in considering that normal rats cannot form pathological scars^11^. The identified mechanism of RHA against scar formation may help to develop a novel and effective pharmaceutical application for fibrosis (eg, scarring) treatment.

## Materials and Methods

### Materials

Di-rhaminolipid with chemical structure in Fig. S1 was a gift from Zijing Bio. Inc. (Huzhou, China) with purity >99%. TGF-β1 was purchased from Peprotech (Rocky Hill, NJ). Collagenase I, Dispase, Calcein-AM, propidium iodide (PI), Alexa Fluor 488 phalloidin, Fura-2-AM, DMEM medium were purchased from Sigma-Aldrich Chemical Company (St. Louis, MO). Primary antibodies (anti-α-SMA and anti-β-Tubulin) and secondary antibodies (goat-anti-mouse, HRP and Alexa Fluor 488) were purchased from Santa Cruz Biotech. Inc. (Dallas, TX). Fetal bovine serum (FBS) was obtained from Gibco (Invitrogen Co. Ltd, Canada). Collagen (type I, from rat tail) was purchased from Biot Biology Inc (Wuxi, China). Sircol insoluble collagen assay kit was purchased from Biocolor Ltd. (Northern Ireland, UK). LDH assay kit was purchased from Saike Biotech. Inc (Ningbo, China). The remaining chemicals were obtained from local chemical suppliers and were all of reagent grade.

### Cell isolation and culture

Normal human foreskin was obtained from patients undergoing surgery, with written informed consent obtained from each patient. The study was performed in accordance with guidelines and regulations of Zhejiang University (Zhejiang, China) and approved by the Ethical and Research Committee of Zhejiang University. Dermal fibroblasts were isolated as described previously^12^. Briefly, dermal layer was obtained from full-thickness skin after dispase treatment at 2.5 U/ml for overnight at 4 °C. The dermal pieces were incubated with collagenase type I at 1000 U/ml for 1 h at 37 °C. The cells were separated by centrifugation at 1000 rpm and then plated into T75 flask with 10 ml DMEM supplemented with 10% FBS, 100 U/ml penicillin and 100 μg/ml streptomycin in a moist atmosphere of 5% CO_2_. Cultures at 80% confluence were harvested and the fibroblasts with 3–5 passages were used in all experiments.

To induce the myofibroblasts, fibroblasts were treated with TGF-β1 at 10 ng/mL in DMEM-0.1% FBS medium for 72 h after 24-h cell starvation in FBS-free DMEM medium. Transformed myofibroblasts that over 95% of the population positively expressedα-SMA was used for further experiments. For detecting the RHA toxicity, fibroblasts and myofibroblasts were separately reseeded on 24 well-plate at density of 1 × 10^5^ cells/well with DMEM medium containing 1% FBS. After incubation overnight, the culture medium was changed by DMEM medium with 0.1% FBS and 0, 10, 20 and 30 mg/L of RHA for 24 h of treatment.

### Assay on cell viability and apoptosis

The Calcein-AM/PI staining was used for direct observation of living/dead cells. The cells were incubated with Calcein-AM/PI solution (4 μM for each fluorescent probe in PBS) for 1 h, and then washed for 3 times in PBS before imaged under a fluorescence microscope (OLYMPUS Ix70) with green/red fluorescent exciters. The cell apoptosis was detected by Annexin V-FITC/PI apoptosis detection kit following the protocol of the kit.

### Assay on cytoplasmic Ca^2+^ concentration, LDH and Calcein leakage

Concentration of intracellular free Ca^2+^ was measured as described previously^13^. Briefly, the myofibroblasts and fibroblasts on 24-well plates were loaded with Fura-2-AM (1 μM) for 1 h at 37 °C. After being washed with Krebs-Ringer-HEPES buffer (108 mM NaCl, 5 mM KCl, 10 mM HEPES, 10 mM D-Glucose, 2 mM MgCl_2_, 2 mM CaCl_2_, at pH 7.4), the myofibroblasts and fibroblasts were incubated with phenol red free DMEM medium with 0.1% FBS at presence or absence of RHA at 10, 20 and 30 mg/L. The fluorescence was recorded at excitation wavelengths of 340/380 nm and the emitted wavelengths of 510 nm (M3, Molecular Devices, USA). At each sampling point, a calculated fluorescence ratio (F340/F380) was determined to represent the Ca^2+^ concentration.

For detecting the Calcein leakage, the myofibroblasts and fibroblasts on 24-well plates were preloaded Calcein by incubating with 4 μM Calcein-AM in culture medium for 1 h at 37 °C. After two washes with Krebs–Ringer–HEPES buffer, the cells were incubated with phenol red free DMEM medium with 0.1% FBS at presence or absence of RHA at 10, 20 and 30 mg/L. The fluorescence in culture medium was recorded at excitation wavelengths of 488 nm and the emitted wavelengths of 535 nm (M3, Molecular Devices, USA).

The lactate dehydrogenase (LDH) leakage assay was following the protocol of the commercial LDH cytotoxicity detection kit^14^.

### Collagen gel contraction assay

Collagen gel lattices were prepared as described previously^15^. In brief, type I collagen (3 mg/mL), 10 × DMEM, and cell suspension (1.2 × 10^6^ cells/mL in DMEM) were mixed on ice in the volume ratio of 9:1:2. 1 mL of the mixture was added to each well of a 12-well culture plate for 0.5 hour at 37 °C. After being solidified, the collagen gels were then free from the sides of the wells and DMEM culture medium containing 0.1% FBS with or without RHA were then added on top of each gel. After 0, 24, 48 and 72 h of incubation, the diameter of each gel was measured three times with a ruler, and the mean value was calculated.

### Determination of soluble and insoluble collagen secreted by myofibroblasts and fibroblasts

After incubated with RHA at 10, 20 and 30 mg/L for 24 h, the culture medium was sampled to detect the soluble collagen secreted by myofibroblasts or fibroblasts. After the rest medium was discarded, insoluble collagen on plate well and inside cells was then collected by conversion of native to insoluble collagen to soluble denatured extraction according to the protocol of Sircol insoluble collagen assay kit (http://www.biocolor.co.uk/site/wp-content/uploads/2016/04/sircol-insoluble-assay.pdf). The collagen contents of soluble and insoluble collagen were both quantified by Sircol collagen assay kit following the protocol, as previously described^16^. The insoluble collagen in each cell culture was also stained by Sirius red for visual observation.

### Fluorescence staining of F-actin and α-SMA in cells

For fluorescence staining, the cells were cultured on the glass slides inside the 35 mm confocal dishes. The F-actin cytoskeleton of cells was stained with Alexa Fluor 488 phalloidin. For immunofluorescence staining, cells were incubated overnight at 4 °C with α-SMA primary antibodies. After three rinses with PBS, incubation with Alexa Flour 488 goat-anti-mouse secondary antibody was performed for 1 hour at room temperature. The nucleolus was stained with DAPI (Vector Laboratories) following the manufacturer’s protocol. The stained cells were imaged by confocal microscopy (Nikon E-1000 + C1 LSCM).

### Western blot analysis

Western blot assay was performed using antibodies specific to α-SMA and β-tubulin. Total cellular proteins were extracted using M-PER reagent from cells, and 20 μg of total protein extract was separated by electrophoresis on 10% SDS-PAGE gels. Proteins were electro-transferred onto PVDF membrane which was then blocked overnight in 5% BSA at 4 °C. Individual membranes were incubated in the PBS-T buffer containing anti-mouse antibodies against α-SMA and β-tubulin followed by 1 h of incubation with a secondary antibody conjugated to HRP. Blots were developed using the ECL staining and observed under a chemiluminescence scanner.

### Rabbit ear scar model

All animal procedures were carried out in accordance with the Guide for the Care and Use of Laboratory Animals by the United States National Institutes of Health. The study was approved by the Ethical and Research Committee of Zhejiang University, China. The rabbit ear scar model was established as previously described^17^. Briefly, 12 adult New Zealand white male rabbits (2.0–2.5 kg) were acclimated and housed under the standard 12 h light/12 h dark cycle with free access of water and diet. To produce a full-thickness wound, rabbits were anaesthetized with 1% pentobarbital (1.5 mg/kg) and then a dermal punch biopsy (7 mm in diameter) was created down to bare cartilage on the ventral surface of each ear. Three punch wounds were made on each ear of the rabbits. 48 hours after surgery, wounded rabbits were randomly divided into three groups. Each wound on the right ear was applied topically with 200 μL of RHA at 0, 1 or 2 g/L in PBS with the solution maintaining for 15 min, while each wound on left ear were similarly treated with 200 μL of PBS. The wound sites were treated once a day for 8-consective-day period and the ear scars were sampled at Day 21 after injury.

### Histologic analysis of ear scar

Scar tissues were fixed overnight in 4% formalin solution and embedded in paraffin. Tissue sections were stained with hematoxylin and eosin (H&E) for morphological assessment. The collagen analysis of skin scars were performed using Masson trichrome stain kit and Sirius red staining^18^. Furthermore, the thin/thick ratio of skin thickness was assessed as scar elevation index (SEI)^19^. The average value was calculated from at least six different sections. All histological measurements were made independently by two observers blind to experimental assignments and the typical results were captured.

### Immunofluorescence staining of α-SMA in ear scar

The paraffin embedded sections were dewaxed and rehydrated through a graduated ethanol series and distilled water. After antigen retrieval by citrate buffer, the sections were blocked with 1.5% fish skin gelatin and then incubated overnight at 4 °C with primary antibody of α-SMA. After three rinses with PBS, incubation with goat-anti-mouse secondary antibody was performed for 1 hour at room temperature. Nuclear staining was performed by mounting medium containing DAPI (Vector Laboratories). Fluorescence microscope (OLYMPUS Ix70) was used to visualize and capture immunostained cells with good resolution.

### Statistical analysis

All data from cell experiments were analyzed by means ± SD from three independent experiments with cells from different strains. Comparisons between multiple groups were performed with the ANOVA test by SPSS, or results from two different groups were tested with the unpaired Student t-test. *P*-values less than 0.05 were considered statistically significant.

## Results

### Myofibroblasts showed higher sensitivity to RHA toxicity than fibroblasts

**Figure 1:**
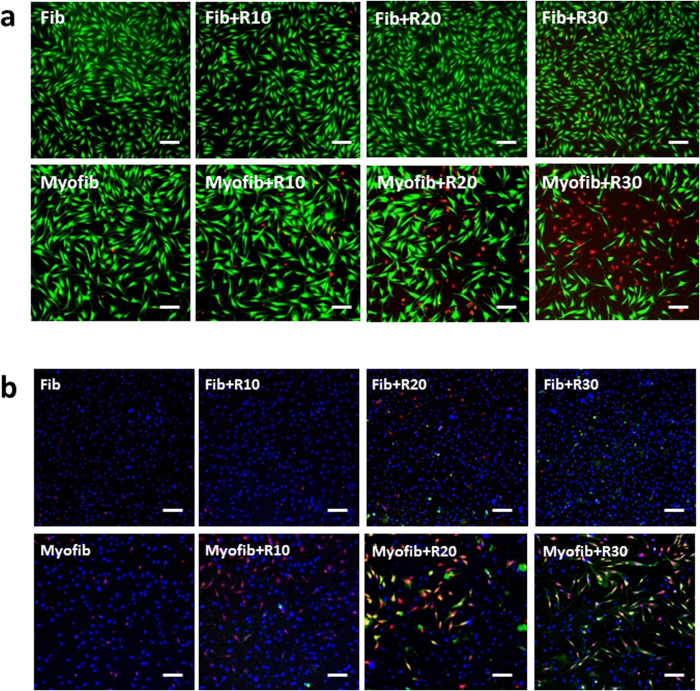
Living/dead cells by Calcein-AM/PI staining and apoptosis assay. (**a**) Alive cells were stained by green Calcein, while dead cells were stained by red PI. (**b**) Apoptotic and necrotic cells were labeled by Annexin V (green) and PI (red) respectively, while the nucleus were stained by Hoechst33342 (blue). Fib = fibroblast, Myofib = myofibroblast, R10, R20 and R30 = di-rhamnolipid at concentrations of 10, 20 and 30 mg/L. Scale bar = 20 μm.

Generally, the tested compounds are co-incubated with fibroblasts before or during the process of TGF-β1 stimulation in anti-fibrotic research *in vitro*^20^,^21^,^22^, aiming to reducing the transformation of myofibroblasts from fibroblasts. In this respect, RHA at 10–30 mg/L was firstly co-incubated with fibroblasts for 12 h before TGF-β1 stimulation, but unexpectedly it could not block the transformation of myofibroblasts from fibroblasts after TGF-β1 stimulation (Fig. S2). To further identify the mechanism of RHA against fibrosis, the fibroblasts were transformed to myofibroblasts by TGF-β1 stimulation before RHA treatment. To our surprise, more than 50% of myofibroblasts died after RHA treatment at 30 mg/L for 24 h while no significant fibroblast death was observed, as reflected by living/dead cell staining using fluorescence dyes of Calcein/PI (Fig. 1a). Correspondingly, the apoptosis detection by Annexin V-FITC/PI/Hoechst33342 staining demonstrated that myofibroblasts exhibited the large amount of apoptotic (green) and necrotic (red) death after RHA treatment at 20–30 mg/L (Fig. 1b).

**Figure 2:**
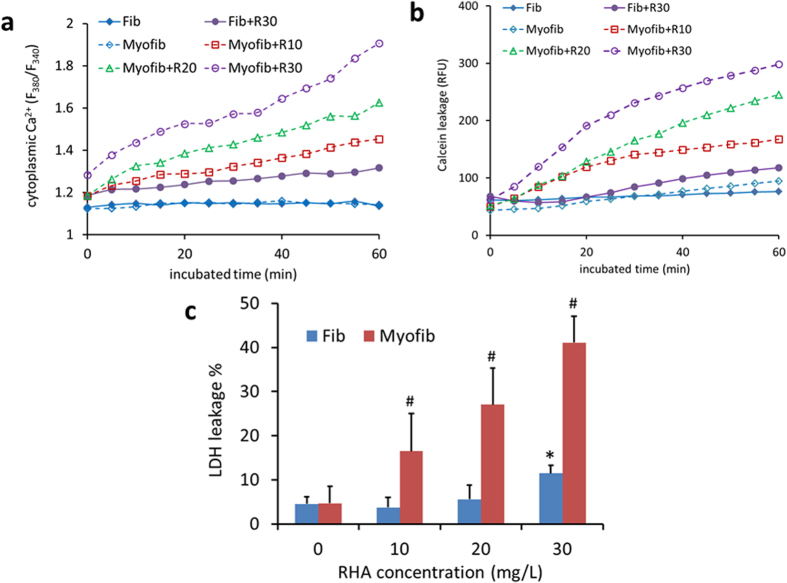
Different toxicity of di-rhamnolipid on fibroblasts and myofibroblasts. (**a**) Cytoplasmic Ca^2+^ alteration during di-rhamnolipid treatment within 1 h. (**b**) Di-rhamnolipid induced leakage of preloaded Calcein within 1 h of incubation. (**c**) LDH leakage of cells after treatment with di-rhamnolipid for 24 h. Fib = fibroblast, Myofib = myofibroblast, R10, R20 and R30 = di-rhamnolipid at concentration of 10, 20 and 30 mg/L. * and ^#^p < 0.05.

The differing toxicity of RHA on the two cells was further confirmed by detecting cytoplasmic Ca^2+^ content as well as the leakage of preloaded Calcein and LDH. Myofibroblasts showed significantly enhanced cytoplasmic Ca^2+^ content (Fig. 2a) and high Calcein leakage (Fig. 2b) immediately after incubation with 10–30 mg/L of RHA. They also exhibited the leakage of LDH (Fig. 2c), a macromolecule, during the prolonged exposure to RHA for 24 h, suggestive of the membrane damage. By contrast, fibroblasts presented little toxic response to RHA treatment even at the high concentration of 30 mg/L (Fig. 2), but exhibited severe toxicity at exposure to RHA over 50 mg/L (Fig. S3). The long-term exposure of dermal fibroblasts to RHA at 50 mg/L for 2 weeks, though has not been detected in our study, only reduced viability by 17% according to previous report^10^. Taken together, RHA might perform anti-fibrotic effect via killing myofibroblasts rather than blocking their transformation.

### RHA inhibited fibrotic indices of myofibroblasts

**Figure 3:**
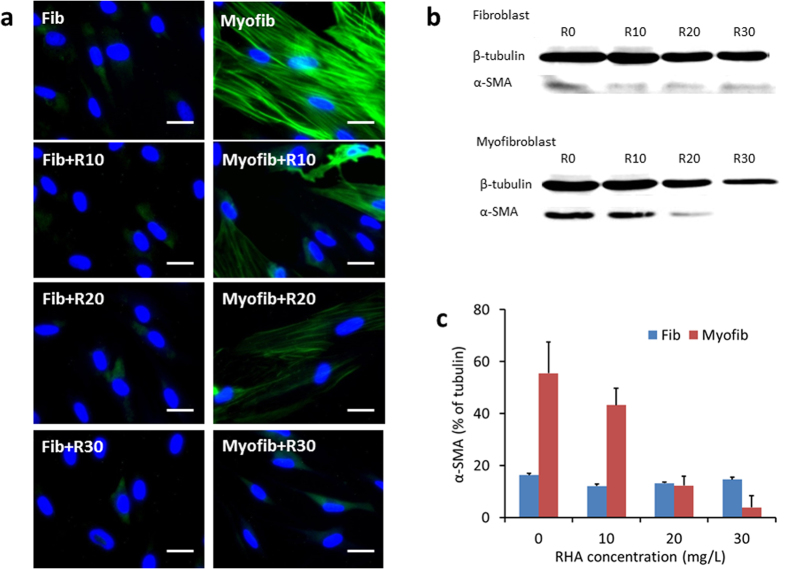
α-SMA expression in fibroblasts and myofibroblasts with or without treatment by di-rhamnolipid. (**a**) Immunofluorescence staining of α-SMA (green). DAPI was used for nuclear counterstaining (blue). Scale bars = 2 μm. (**b**) α-SMA expression by Western blotting. (**c**) Data digitized from images of Western blotting by Image Pro Plus 6.0. Fib = fibroblast, Myofib = myofibroblast, R10, R20 and R30 = di-rhamnolipid at concentration of 10, 20 and 30 mg/L.

The fibrotic indices of myofibroblasts, characterized by α-SMA expression, collagen secretion and contraction, were then detected to confirm the potential anti-fibrotic effect of RHA. As expected, RHA greatly suppressed the α-SMA expression in myofibroblasts according to immunofluorescence staining (images in Fig. 3a and quantified by Image J in Fig. S4) and Western blotting assay (Fig. 3b and c). Naturally, α-SMA was not significantly expressed in fibroblasts.

**Figure 4:**
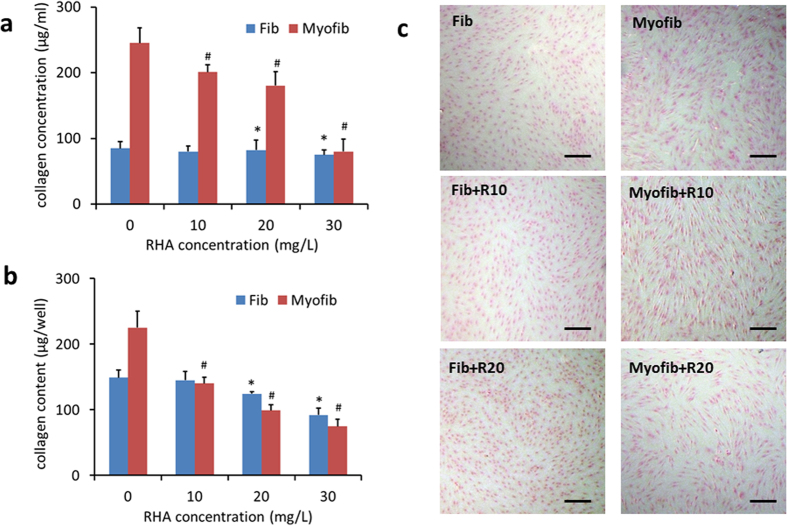
Collagen secretion by fibroblasts and myofibroblasts after di-rhamnolipid treatment. Soluble collagen concentrations in culture medium (**a**) and insoluble collagen content on plate well (**b**) assayed by Sircol collagen assay kit. * and ^#^p < 0.05. (**c**) Sirius red stained insoluble collagen secreted by cells. Scale bar = 20 μm. Fib = fibroblast, Myofib = myofibroblast, R10, R20 and R30 = di-rhamnolipid at concentration of 10, 20 and 30 mg/L.

**Figure 5:**
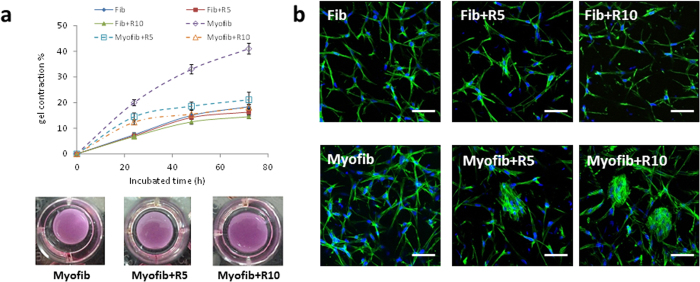
Contraction of collagen gel lattices by fibroblasts and myofibroblasts after di-rhamnolipid treatment. (**a**) Time-dependent gel contraction (above) and gel morphology at 48 h (below) after di-rhamnolipid treatment. ^#^p < 0.05. (**b**) F-actin morphology of fibroblasts and myofibroblasts entrapped inside the collagen gel. F-actin was stained green by Alexa Fluor 488 phalloidin, while nucleus were stained blue by DAPI. Scale bar = 10 μm. Fib = fibroblast, Myofib = myofibroblast, R5 and R10 = di-rhamnolipid at concentration of 5 and 10 mg/L.

Both myofibroblasts and fibroblasts secreted soluble and insoluble collagen, but myofibroblasts normally synthesized collagen 1–2 folds higher than fibroblasts (Fig. 4). However, upon RHA treatment, myofibroblasts showed the reduced collagen synthesis at an equivalent level as fibroblasts (Fig. 4a and b, and sparsely distributed Sirius red at light color in Fig. 4c and Fig. S5). The contractile activity of the two cells with or without RHA treatment was further reflected in collagen gel lattices. Myofibroblasts did cause more gel contraction than fibroblasts without RHA treatment (Fig. 5a) but showed the similar gel contractibility as fibroblasts under RHA treatment even at a low concentration of 10 mg/L (Fig. 5a). According to the F-actin staining, RHA treatment for 48 h turned collagen gel-entrapped myofibroblasts into aggregates (Fig. 5b) while did not alter the elongated morphology of fibroblasts, indicative of the more adverse effect on cytoskeleton of myofibroblasts.

### RHA reduced the hypertrophic scar formation on rabbit ear

**Figure 6:**
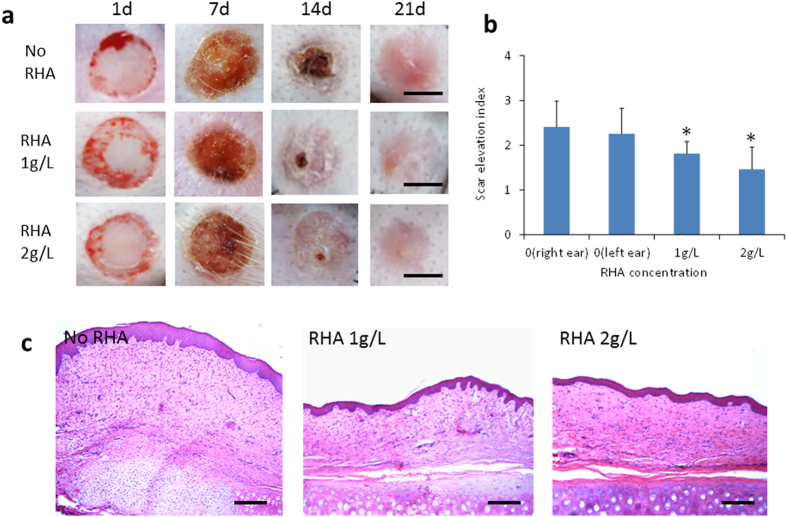
Di-rhamnolipid inhibits scar formation on rabbit ear. (**a**) Representative images of skin wounds at different time points. Scale bar = 5 mm. (**b**) Scar elevation index (SEI) in di-rhamnolipid treated and untreated groups at 21 days post-wounding. *p < 0.05. (**c**) Histological staining of scars at 21 days post-wounding. Scale bar = 100 μm.

The finding in cell cultures suggested that RHA might have anti-scarring effect *in vivo*. As known, rabbit ear model behaves like human wounds in both scarring mechanism and response to scar resisting drugs^17^, while most of animals such as rats cannot form pathological scars and thus are not suitable for scarring investigation^11^. Thus, we further verified the anti-scarring effect of RHA on rabbit ear model. In RHA-treated groups, each rabbit ear wound was treated by RHA at doses of 1 and 2 g/L, respectively, in considering that the RHA concentrations in wound tissue (sample size of 5 × 5 mm) were detected within the range of 10 to 35 μg/g (data not shown), very close to the doses in cell culture at 10–30 mg/L. At postoperative Day 7, RHA showed a dose-dependent inhibition on wound suppuration (Fig. 6a), which might facilitate the wound healing. Although all the wounds on ears were totally reepithelialized on gross examination within Day 14, the untreated ear wounds had a stiff and visibly raised scar at Day 21 (Fig. 6a) while the RHA-treated ear wounds showed less visible and softer scars (Fig. 6a). On postoperative Day 21, H&E staining showed that the untreated scars were obviously thick and of damaged cartilage structure. However, the RHA treatment sections appeared flatter and thinner (Fig. 6c), in consistent with the gross macroscopic observation. Similar with the result of H&E staining, the RHA treated scars had the much lower scar elevation index (SEI) than the controls (Fig. 6b). Moreover, RHA treatment at a high dose of 5 g/L, though elicited no side effect, did not enhance the therapeutic effect compared to that at 2 g/L (data not shown).

**Figure 7:**
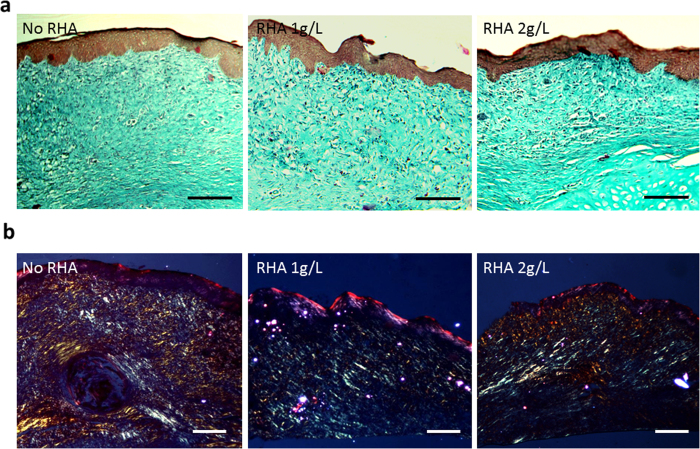
Effect of di-rhamnolipid on collagen fiber morphology in rabbit ear scars. (**a**) Masson staining and (**b**) Sirius red staining of collagen in scar tissue at 21 days post-wounding. Scale bar = 100 μm.

Masson staining was used to evaluate the effects of RHA on producing and remodeling collagen fibers. As found, collagen disorderly distributed in scars of the control group at a less mature mode, while were more orderly arranged at parallel to the epithelium in RHA treatment scars, which was particularly noticed in the 2 g/L RHA treated group (Fig. 7a). The distribution of collagen type in scar tissue was further examined post to Sirius red staining under the polarized light microscope, whereas type I collagen and type III collagen respectively showed the colors of red/yellow and green^18^. As shown in Fig. 7b, type III collagen dominated the extracellular matrix in RHA treated scars, consisting with the high ratio of type III collagen in scarless healing of fetus^23^. By contrast, type I collagen mainly accounted for the matrix on the control group, showing a similar distribution as that in adult skin healing^23^.

**Figure 8:**
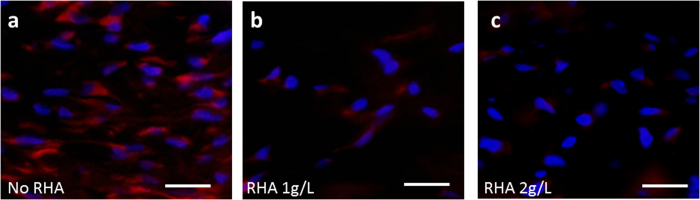
Immunofluorescence staining of α-SMA (red) on rabbit ear scar section at 21 days postwounding with or without di-rhamnolipid treatment. DAPI was used for nuclear counterstaining (blue). Scale bar = 10 μm.

The immunofluorescence staining of α-SMA in the testing groups was shown in Fig. 8. Abundance of α-SMA positive cells were found in the section of control group (stained red, Fig. 8a), indicating the persistence of myofibroblasts, while the α-SMA expression was remarkably attenuated in RHA treated group (Fig. 8b and c, and quantified by Image J in Fig. S6).

## Discussion

Cell cultures facilitated the mechanistic investigation, illustrating that RHA killed myofibroblasts without causing significant toxicity to normal dermal fibroblasts (detected in our study) and keratinocytes (previously reported)^10^. By contrast, *in vivo* rabbit ear model provided convincible evidences for proposing the potential application in scar therapy, and thus has been widely used to evaluate the effects of anti-scarring compounds^24^,^25^. The anti-fibrotic effect of RHA, though has been reported on burn wounded rats^9^, was not convincing as our study on rabbit ear model because rat skin reflected the fibrosis by the sole index of collagen content in healed tissue^9^. Differing from rats or cell cultures, rabbit ear model reflected not only the reduced scar elevation by RHA but also the altered ratio of collagen types in scars. Besides the mechanism discovered in cell culture, RHA might also reduce the scar formation via its anti-infective effect since the cartilage infection has been completely suppressed by RHA in rabbit ear models (Fig. 6c). However, the detailed effects of RHA on suppression of inflammation^9^, anti-microbial^26^ and as immunomodulators^27^,^28^ could not be uncoupled in the complex and well-orchestrated process of wound healing and scarring. In addition, the anti-fibrotic effect of RHA on female animals still needs further confirmation, since only male cells and rabbits were used in our study.

The targeted toxicity of RHA on myofibroblasts might be related to the high stiffness of myofibroblasts for their expression of contractile fibers^1^. The plasma membrane of such rigid cells were likely to be damaged via membrane blebbing under a mechanical force on cortical layer or plasma membrane^29^. To be even worse, rigid cells were more difficult to reseal their membrane damage than cells with less stiffness^30^. As RHA could insert into and perturb the lipid-bilayer membrane^31^, we assumed that RHA might injury more of rigid myofibroblast membrane, causing higher toxicity. This hypothesis was likely supported by Fig. 2 which showed the high Ca^2+^/Calcein/LDH leakage from myofibroblasts after RHA treatment. Nevertheless, the mechanism of RHA toxicity on myofibroblasts needs further investigation.

As a natural product synthesized by bacteria, RHA has been enthusiastically proposed for use in cosmetics and pharmaceutics due to its low toxicity and biodegradability^32^. The external application of RHA on animal skin seemed to be safe according to our observation on rabbit ear wound (Fig. 6a) and previously case on burn wounded rats^9^. RHA has even been applied to cure the decubitus ulcer of patient^33^, further indicating its safety in human external application. Moreover, RHA also showed high safety in oral administration (ED_50_ > 5000 mg/kg)^34^ and subcutaneous injection (low toxicity at 120 mg/kg per day)^9^ in rodents. Actually, RHA has been approved by FDA for use in fruit, vegetable, and legume crops for their low acute mammalian toxicity and nonmutagenicity^35^. In this regard, RHA will be possibly developed as an anti-scarring drug for potential applications in the future.

## Conclusion

RHA, a biosurfactant secreted by bacteria, has potent effect against scar formation via a unique mechanism of targeted killing of myofibroblasts. In cell culture system, RHA elicited more toxicity to myofibroblasts transformed from fibroblasts by TGF-β1 stimulation and inhibited the fibrotic indices of α-SMA expression, collagen secretion and contraction. This anti-fibrotic effect of RHA was further proved on rabbit ear hypertrophic scars by reducing the scar elevation index, collagen distribution and α-SMA expression in scar tissues. The finding in this paper may help to develop a novel and effective pharmaceutical applications for scar therapy.

## Additional Information

**How to cite this article**: Shen, C. *et al*. Targeted killing of myofibroblasts by biosurfactant di-rhamnolipid suggests a therapy against scar formation. *Sci. Rep.* **6**, 37553; doi: 10.1038/srep37553 (2016).

**Publisher's note:** Springer Nature remains neutral with regard to jurisdictional claims in published maps and institutional affiliations.

## Supplementary Material

Supporting Data
