# Supplementary material for: Targeted killing of myofibroblasts by biosurfactant di-rhamnolipid suggests a therapy against scar formation
Source: Sci Rep. 2016 Nov 30;6:37553. doi: 10.1038/srep37553 (PMC5128858; doi:10.1038/srep37553)
Supplement: Supporting Data [file srep37553-s1.pdf]

Supplementary data

Targeted killing of myofibroblasts by biosurfactant di-rhamnolipid suggests a therapy against scar formation

Chong Shen[1], Lifang Jiang[1], Huawei Shao[2], Chuangang You[2], Guoliang Zhang[3], Sitong Ding[1], Tingwei Bian[1], Chunmao Han[2], Qin Meng[1,*]

Fig S1

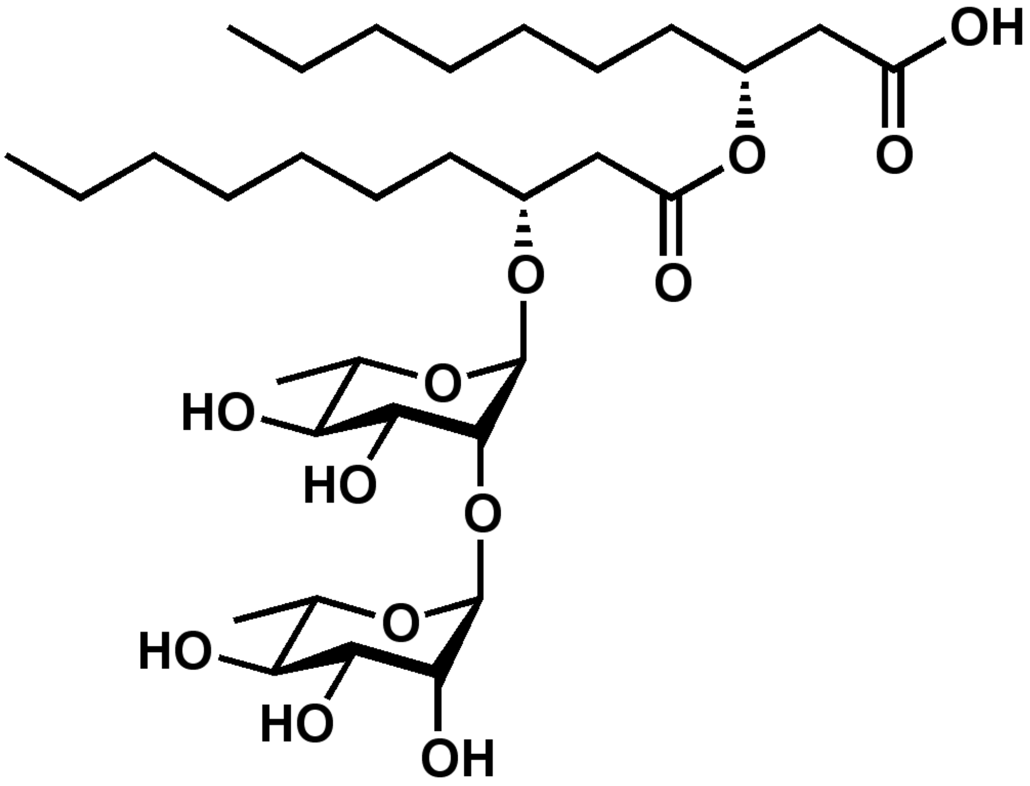

Fig S1. Chemical structure of di-rhamnolipid.

Fig S2

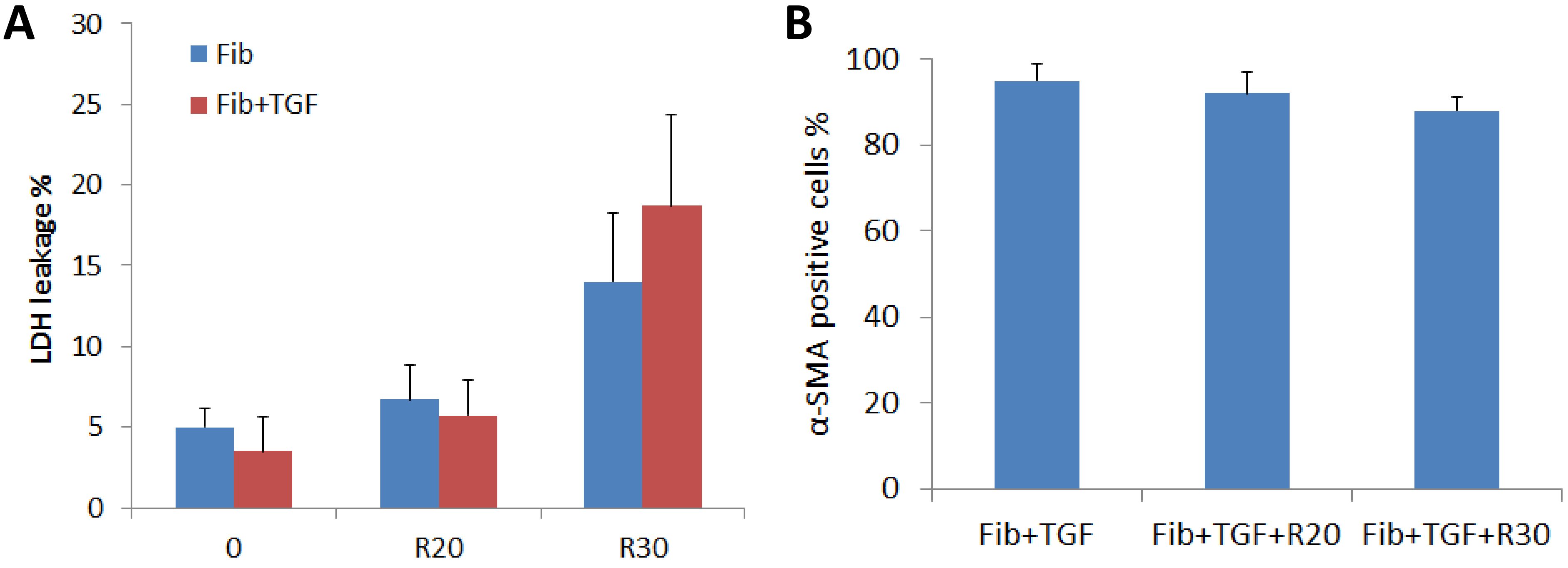

Fig S2. Fibroblasts were cotreated by RHA and TGF-β1. (A) RHA toxicity on fibroblasts with or without TGF-β1 treatment. (B) percent of α-SMA positive cells after TGF-β1 stimulation.

Fig S3

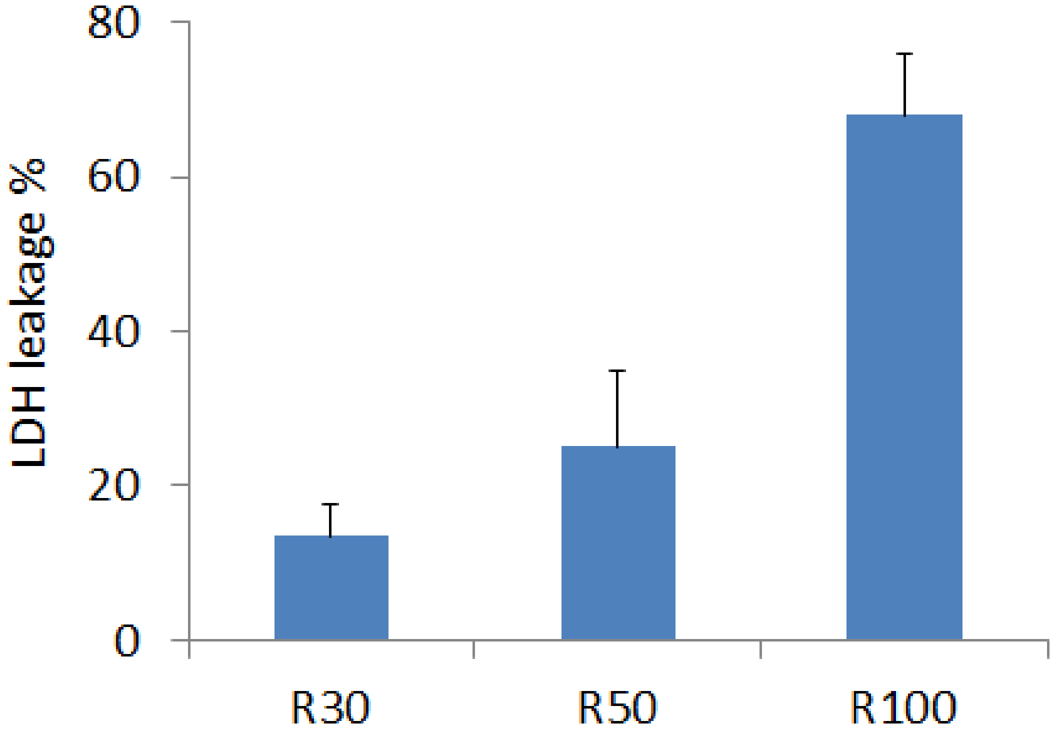

Fig S3. LDH leakage of fibroblasts after treatment with RHA at 30, 50 and 100 mg/L for 24 h.

Fig S4

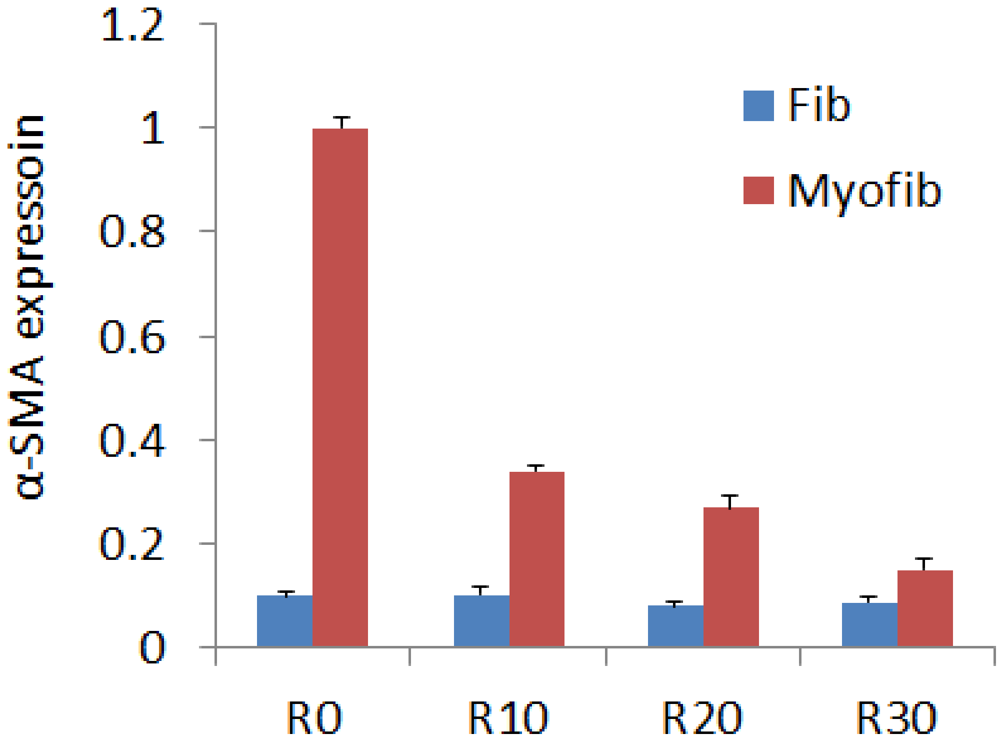

Fig S3. The α-SMA expression after RHA treatment in Fig 3A was quantified by Image J.

Fig S5

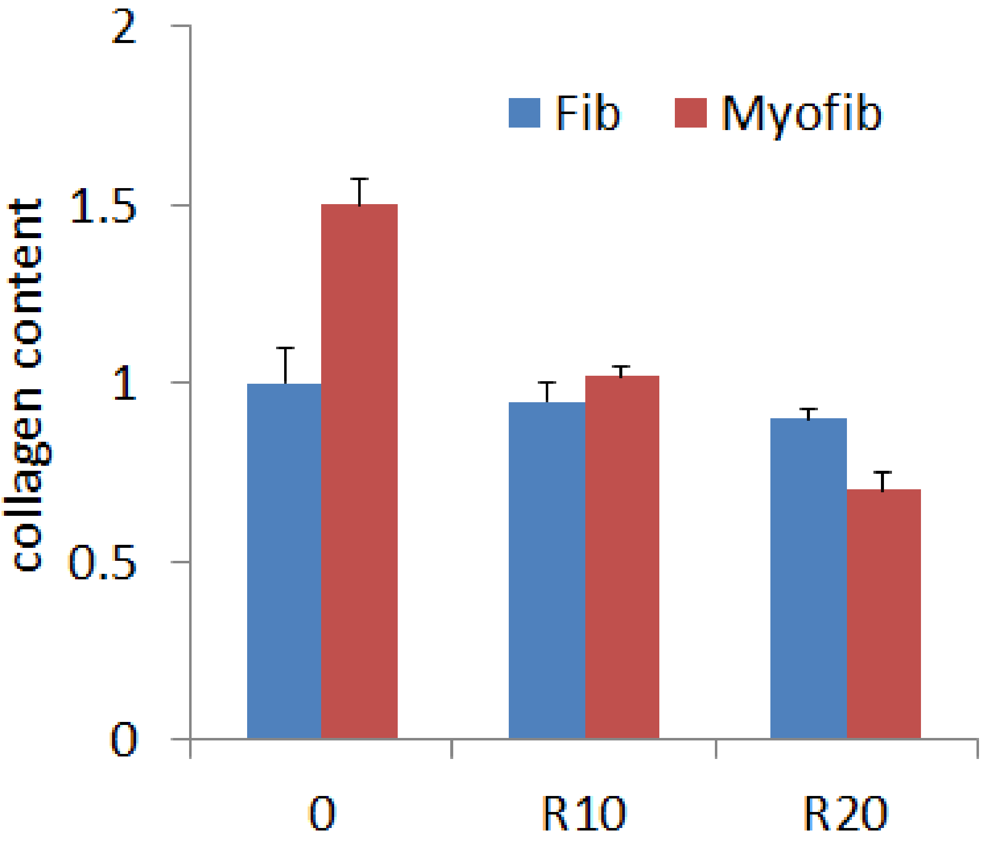

Fig S5. The collagen content after RHA treatment in Fig 4c was quantified by Image J.

Fig S6

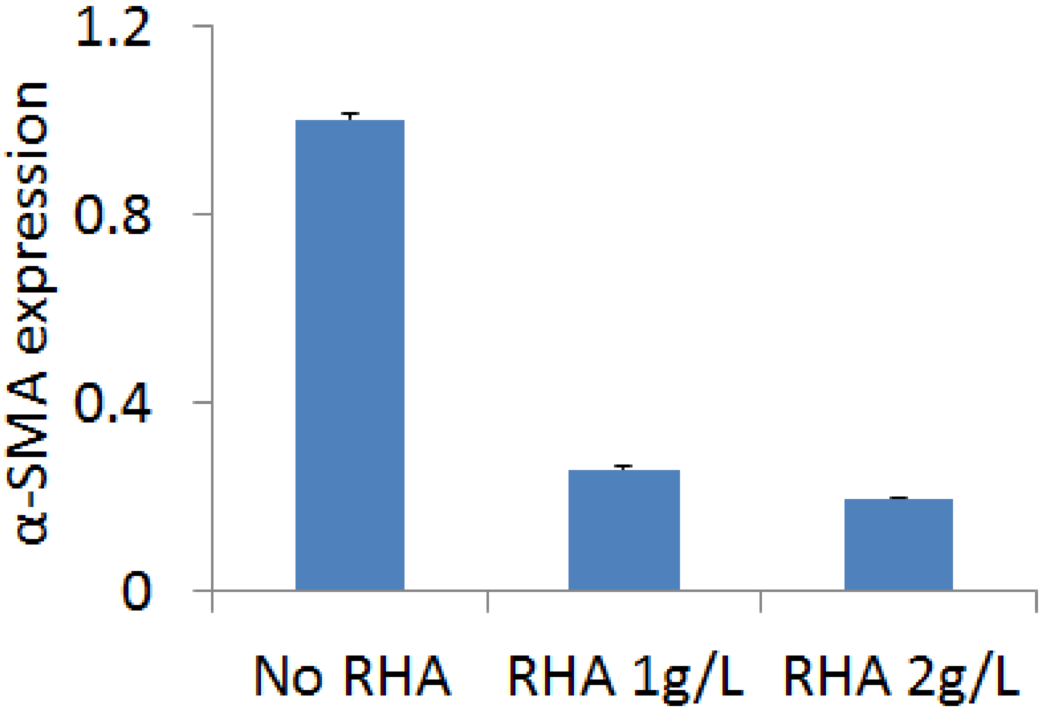

Fig S6. The α-SMA expression after RHA treatment in Fig 8 was quantified by Image J.
